# The status, or conspicuous absence, of regulation of proprietary or locally manufactured African traditional medicines in South Africa: a constitutional injustice

**DOI:** 10.3389/fpubh.2026.1907885

**Published:** 2026-08-20

**Authors:** Aisha Gambo, Nceba Gqaleni, Limakatso Lebina

**Affiliations:** 1 Sub-Discipline of Traditional Medicine, School of Medicine, College of Health Sciences, University of KwaZulu-Natal, Durban, South Africa; 2 Africa Health Research Institute, Durban, South Africa

**Keywords:** African Traditional Medicines (ATMs), Category D complementary medicines, constitutional injustice, regulatory framework, South African Health Products Regulatory Authority (SAHPRA)

## Abstract

Despite heavy reliance on African Traditional Medicines (ATMs) by approximately 80% of Black South Africans, South Africa exhibits a conspicuous regulatory gap characterized by a complete absence of an official regulatory framework for ATMs indigenous to its borders. On the contrary, the South African Health Products Regulatory Authority (SAHPRA) provides a comprehensive regulatory framework for non-indigenous complementary medicines under Category D. This policy and practice review argues that this unambiguous dichotomy constitutes a structural and constitutional injustice. It violates constitutional rights to equality and healthcare access and exposes consumers to significant public health and economic risks. Using a document analysis, this study examines SAHPRA’s current guidelines and relevant legislation and found no evidence of ATM regulation. To rectify this disparity, we propose a simplified, layered, or risk-based model like those successfully implemented in other African nations such as Ghana, Nigeria, Uganda, and Lesotho. These ensure cultural respect, consumer safety, and legal equity within the national healthcare system.

## Introduction

### Traditional medicine in South Africa

The World Health Organisation (WHO) advances the recognition, acceptance, and evidence-based integration of Traditional Medicine (TM) into the health systems of its member states, including South Africa, as stated in the Global Traditional Medicine Strategy 2025–2034 (1, 2). The South African Constitution, through the Bill of Rights, assures citizens the right to equality, to participate in their chosen cultural life, and to select a particular healthcare provider or facility for treatment (3). Therefore, effective integration of TM into the national health system can only be achieved when regulatory frameworks are established and upheld in a manner that respects South Africans’ cultural and religious beliefs.

Regardless of these mandatory constitutional obligations, traditional medicine has not been incorporated into South Africa’s national healthcare system, which remains grounded in conventional or allopathic medicine. Traditional medicine continues to serve as an essential alternative practice, with approximately 80% of Black South Africans depending on it (4, 5). Although the African traditional medicine system is poorly documented, it is recognized as one of the oldest and most diverse medical systems globally, interweaving African traditional healing with cultural practices and religious beliefs to provide holistic healthcare (6).

South Africans utilise traditional medicine primarily due to cultural practices and a personalized, client-centered approach to healthcare. Additionally, TM addresses spiritual and emotional aspects, attending to social and spiritual concerns that are significant in African cultures. Its accessibility has made it a popular choice among people living with HIV (PLHIV) undergoing antiretroviral therapy (ART). Other conditions treated with TM in South Africa include respiratory diseases, tuberculosis, urinary tract infections, and sexually transmitted infections (STIs) (6, 7). These factors, alongside the limited access to orthodox practitioners and dissatisfaction with conventional medicine, are reasons for the continued use of TM, especially among rural communities (1, 4). Furthermore, the significant economic impact of the traditional medicine trade, projected at R20 billion per year (8), emphasises its societal importance.

In South Africa, herbal medicines are the most consumed traditional medicines. Users purchase these products from traditional health practitioners, herbal markets and pharmacies. They are formulated as preparations packaged in recyclable bottles or paper, with labels often not disclosing the full ingredient lists. Despite several initiatives adopted to improve the safety, efficacy, labelling, and traditional use of herbal medicines, the safety and quality of many products remain unknown, as they are not registered with the South African Health Products Regulatory Authority (SAHPRA) (9). In 2005, Nicoli Nattrass argued that there is a disproportionate concentration among lower-income and less-educated sectors of the Black South African population who rely on traditional medicine as a means of healthcare (10). This perspective has been frequently disputed by contemporary findings (6, 11). Regardless of the demographic profile of the user, the current lack of safety and quality control measures leaves millions of citizens vulnerable to unregulated products and health risks.

Critically, the status of ATM regulation and registration in South Africa is not merely underdeveloped; it is non-existent. The South African Health Products Regulatory Authority regulates complementary medicines across six main disciplines: aromatherapy, ayurveda, homeopathy, traditional Chinese medicine, Unani Tibb, Western herbal medicine, and combination products. According to SAHPRA, a complementary medicine is any substance or mixture of substances that originates from plants, fungi, algae, seaweeds, lichens, minerals, animals, or other substances as determined by the authority; it is used or purportedly suitable for use in maintaining, complementing, or assisting the physical or mental state; or to diagnose, treat, mitigate, modify, alleviate, or prevent disease or illness; and it is used as a health supplement or in accordance with disciplines determined by the authority (12).

The WHO defines Complementary Medicine as a broad set of healthcare practices that are not part of a country’s traditional or conventional medicine and are not fully integrated into the established healthcare system (13). In contrast, traditional medicine refers to the use of indigenous practices unique to different cultures to maintain health and prevent, diagnose, improve, or treat physical and mental illnesses based on theories, beliefs, and experiences (14).

Applying the WHO’s definitions, the term ‘complementary medicine’ in the South African context refers to all other TM that are not indigenous to South Africa (6). While Complementary Medicines are indeed Traditional Medicines, they are not considered ATM. A comprehensive regulatory framework for complementary medicines has been established in South Africa, yet no such framework exists for African Traditional Medicine (ATM), which are the medicines that are indigenous to the country.

This policy and practice review examines this regulatory anomaly and argues that it constitutes a constitutional and structural injustice that undermines both constitutional rights and public health. Our argument is provided using a progressive format as follows: (1) the specific injustice or regulatory harm is defined; (2) evidence that such harm exists in the absence of ATM regulation is provided; (3) precisely what is lacking in the current South African system is identified; (4) a regulatory solution is proposed.

### Defining the specific injustice and harm

Constitutional Injustice refers to a situation where actions or omissions by a state violate the rights that are entrenched in its constitution (15). In South Africa, the Bill of Rights guarantees every citizen the right to equality, dignity, and access to healthcare. Furthermore, it protects the right of persons to participate in the cultural life of their choice. The state, through SAHPRA, has a constitutional obligation to protect the health and wellbeing of all its citizens (3). By diligently creating a regulatory framework for foreign traditional medicines while failing to create one for indigenous ATMs, the state is providing preferential treatment to foreign medical systems. This creates a dichotomous system where the users of Ayurveda, Traditional Chinese Medicines, or Western Herbal Medicine have their safety and quality duly evaluated by the state, while the approximately 80% of Black South Africans who depend on ATM are left without any official consumer protection. This directly violates the constitutional right to equality and the right to access healthcare that has been demonstrated to be safe (3).

Furthermore, there is evidence of structural injustice that is rooted in the current regulatory system. Structural injustice is defined as the presence of a systemic outcome that is embedded in the policies and procedures of the state institution that systematically disadvantages a particular group (16, 17). This results in a system that, despite being facially neutral, produces discriminatory results. The regulatory gap is an example of this, whereby the state has a working system for registering and regulating non-indigenous medicines. This system is functional and detailed. The complete absence of a parallel system for ATM is not due to a lack of capacity, as capacity has been demonstrated to exist. It is the result of a structural decision, whether conscious or unconscious, to prioritize non-African health systems. This is a form of structural inequality and stigma that undermines the dignity of the Black majority as it signals that their traditional healthcare practices and cultural beliefs are unworthy of state protection.

These injustices, attributable to a complete absence of a regulatory framework for African Traditional Medicine (ATM), expose a significant majority of Black South Africans who rely on these remedies for their healthcare needs to substantial public health and economic risks (6, 18). Without appropriate quality control measures, adequate labeling, and suitable patient information, the safety, efficacy, and purity of these herbal medicines are severely compromised. This lack of regulation directly fosters patient safety hazards, as unregulated products may be contaminated with toxic heavy metals, aflatoxins, or microbial pathogens, or may contain incorrect plant species leading to toxicity (19, 20). Furthermore, the absence of a mandatory pharmacovigilance system prevents the effective monitoring and reporting of adverse drug reactions (21, 22). This creates critical risks regarding herb-drug interactions for millions of individuals undergoing chronic treatments such as antiretroviral therapy (ART) or tuberculosis care. Beyond these public health concerns, the unregulated nature of the multibillion-rand traditional medicine market facilitates widespread economic exploitation and consumer fraud (8). In the absence of mandatory product registration and marketing standards, manufacturers can propagate unsubstantiated therapeutic claims with impunity and distribute adulterated or counterfeit goods, leaving vulnerable consumers unprotected against exploitation (23).

Having established the injustices and the harms that could exist due to this regulatory anomaly, the need for a regulatory framework for ATM in South Africa cannot be overemphasized.

### Study design, data collection and analysis

Document analysis was used to select official and legal documents from the SAHPRA website, which is the drug regulatory authority in South Africa. Other relevant documents addressing the research topic from additional sources were also identified. Document analysis was used for data selection and data collection. Document analysis is a qualitative research method in which the researcher analyzes and interprets documents to provide meaning and understanding to a research topic (24). Data selection was divided into two categories based on the study aim: (1) regulation of ATM and complementary medicines in South Africa and (2) registration of ATM and Complementary Medicines in South Africa. Data were entered into Microsoft Excel 2505.

## Results

It must be stated clearly that we found no evidence of a regulatory framework for African Traditional Medicines (ATMs) under SAHPRA. Instead, we found extensive documentation on the regulation of complementary medicine. We cannot ascertain if this is a result of international hegemonic policy influence, most notably those endorsed by the World Health Organization (WHO) over its member states (South Africa included) (25). In the following section, we describe the status of complementary medicine regulation in South Africa. We are unable to determine whether this framework serves as a proxy for ATMs, a question that remains unsolved for the industry, researchers, and consumers alike.

### Status of regulation of African Traditional Medicine (ATM) in South Africa

The South African Health Products Regulatory Authority (SAHPRA) is a unit of the National Department of Health. It was created to ensure optimum health and wellbeing in humans and animals. SAHPRA is a Schedule 3A public entity responsible for regulating health products for human and animal use, licensing manufacturers, wholesalers, and distributors of medicines, medical devices, and other health-related devices, and ensuring that clinical trials adhere to ethical standards (26).

The SAHPRA board and executive management team are responsible for overseeing the regulation of all health products in South Africa, including complementary medicines (26). The authority is guided by the Medicines and Related Substances Act of 1965 and Hazardous Substances Act of 1973.

Amendments to the general regulations concerning the Medicines and Related Substances Act, 1985 (Act 101 of 1965) were published by the Minister of Health on the 15th of November 2013. These amendments instituted a new category of medicines, Complementary Medicines (Category D), and a regulatory framework for this category was effectively established (12).

SAHPRA regulates complementary medicines across six main disciplines: The recognised disciplines include Aromatherapy, Ayurveda, Homeopathy, Traditional Chinese Medicine, Unani Tibb, Western Herbal Medicine, and combination products. This category was expanded following extensive interaction with stakeholders, resulting in two subcategories of Category D. This is reflected in the General Regulations published under Government Notice 859 in the Government Gazette 41,064 on August 25, 2017. Critically, these new subcategories accommodate disciplines of traditional medicine that are not indigenous to South Africa (discipline-specific medicines) and more modern supplement-type medicines (health supplements) (12). African Traditional Medicines, which are indigenous to South Africa, are notably absent.

The Medicines and Related Substances Act, 1965 (Act 101 of 1965), stipulates that quality, safety, and efficacy will guide the registration and availability of complementary medicines, consistent with Section 1(2) of the Medicines Act and in accordance with their relative risk.

### Registration of complementary medicines

Discipline-specific medicines are classified as high- or low-risk. High-risk medicines require clinical evidence to justify their safety and efficacy, whereas low-risk medicines may rely on traditional evidence. Classification is primarily based on indications, composition, and dosage forms. Health supplements are limited to low-risk substances and indications, as specified in the SAHPRA guidelines, and must comply with specific lists of substances, dosage ranges, and indications (12, 23).

Registration of all complementary medicines that fall under Category D took effect on 15 November 2013 by the then Medicines Control Council (now SAHPRA), by virtue of powers vested by section 14(2) of the Act and approved by the Minister of Health. The Act permitted continued rights of sale for complementary medicines, provided that an application was submitted for their registration by the prescribed deadlines; they were manufactured, imported, exported, wholesaled, or distributed by a licence holder; they were compliant with the requirements of specific sections and regulations of the Medicines Act; and they were indicated for low-risk purposes, which included general health enhancement without reference to specific diseases, health maintenance, or relief of minor symptoms not related to a disease or disorder (12).

### Use of the ZA-CTD format for registration applications

Registration applications for complementary medicines must be submitted in the South African Common Technical Document (Za CTD) or South African Electronic Common Technical Document (Za eCTD) format, which are both organised into five modules. The modules are as follows: Administrative Information; Common Technical Summaries; Quality; Non–Clinical study reports, and Clinical study reports (27, 28).

Table 1 shows the criteria for determining the safety of the indications and health claims. The indications are classified into two risk levels: high- and low-risk indications or claims. It is mandatory to consult the risk levels, various types of claims, and required evidence when determining the criteria for the safety of indications and health claims, as provided in Table 1 (23).

**Table 1:** Risk level, type of claim, and evidence required.

Risk level	Type of claim	Claim evidence required to support the claim
Low risk	• General health enhancement without any reference to specific diseases • Health maintenance, including nutritional support. • Relief of minor symptoms (not related to a disease or disorder).	• Clinical data to be evaluated AND/OR: • Two of the following four sources that demonstrate adequate support for the indications claimed: 1 Recognised Pharmacopoeia 2 Recognised Monograph 3 Three independent written histories of use in the classical or traditional medical literature or 4 Citations from other *in vivo*, *in vitro* studies, case reports, or others.
High risk	• Treats/cures/manages any disease/disorder. • Prevention of any disease or disorder. • Reduction of risk of a disease/disorder. • Aids/assists in the management of a named disease/disorder or sign/symptom of a named disease/disorder. • Relief of symptoms of a named disease or disorder. • Treatment of proven vitamin or mineral deficiency diseases.	• Clinical data to be evaluated AND • Two of the following four sources that demonstrate adequate support for the indications claimed: 1 Recognised Pharmacopoeia 2 Recognised Monograph 3 Three independent written histories of use in the classical or traditional medical literature, or 4 Citations from other in vivo, in vitro studies, case reports, or others.

Table 2 summarizes the requirements for documenting data on safety, efficacy, and quality for the registration of complementary medicines in South Africa. Likewise, the safety requirements for post-marketing data and the unacceptable presentation using misleading or confusing information regarding the content or proper use of the medicine on any aspect of the product presentation are provided in Table 2 (23, 29).

**Table 2:** Summary of the requirements for documentation of data on safety, efficacy, and quality for the registration of complementary medicines in South Africa.

Requirements	
Safety	The safety documentation should include: • overview of safety • any studies that address specific safety issues • reports (where possible) of adverse effects reported to the National Adverse Drug Event Monitoring Centre • reports of adverse effects from accepted international sources • post-marketing data. Full evidence of tissue residue data of products that have been used in animals destined for human consumption must be included.
Post marketing data	- All data on the worldwide marketing experience, including all relevant Post Marketing data available. This may include published and unpublished data. - Any new or different safety issues identified following marketing and afterwards should be highlighted, and any regulatory action taken in relation to safety by a regulatory agency abroad should be detailed. - Details of the number of people estimated to have been exposed should be provided and categorised, as appropriate, by indication, dosage, route of administration, treatment duration, and geographical location. - A record of adverse reactions should be collected, collated, and maintained after they have been reported for the registered product, and this should be available for inspection to the SAHPRA in accordance with the ADR guideline (Reporting Adverse Drug Reactions in South Africa).
Efficacy	Table 1 should be referred to determine the type of evidence needed to authenticate a given claim. The criteria to be considered in the evaluation of efficacy for all complementary medicines may include: • established traditional use, • pre-clinical data and • evidence from clinical trials in animals and human beings • references appropriate for the risk level of the associated claim.
Quality	The types of information and level of detail depend on the active ingredient(s) and on the risk associated with the product. Information that should be provided includes: • the substance name, • composition, • structure (chemical and/or morphological, where possible) and general properties •manufacturing details, including process and controls •substance characteristics, including impurities and incidental constituents •specifications and details of analytical test methods, with method validation data •stability data.
Unacceptable presentation	The presentation (including package inserts, patient information leaflets, labelling, and packaging) of complementary medicines is not acceptable if it is capable of being misleading or confusing as to the content or proper use of the medicines. Proper translation of languages must be ensured. In addition, the presentation of complementary medicines is unacceptable: • if the words “natural” and “gentle” are used to imply safety • if it states or suggests that the product has ingredients, components, or characteristics that it does not have; • if a name applied to the product is the same as the name applied to other products that are already supplied in South Africa, where those other products contain additional or different therapeutically active ingredients; • if the label of the product does not declare the presence of a therapeutically active ingredient; • if a form of presentation of the product may lead to unsafe use of the product or suggest a purpose that is not in accordance with conditions applicable to the sale of the product in South Africa or could be confused with an existing registered or unregistered “brand.”

## Discussion and actionable recommendations

While Complementary Medicines are Traditional Medicines, they are not African Traditional Medicines (ATMs). Owing to the complete absence of evidence of ATM regulation by the South African Health Products Regulatory Authority (SAHPRA), this study instead reports on the historical evolution of SAHPRA on the general Regulations under the Medicines and Related Substances Act, 1985 (Act 101 of 1965). This led to the effective establishment of a robust regulatory framework for Category D complementary medicines (12). It is a profound anomaly, indeed, a constitutional injustice that South Africa has created a regulatory pathway for non-indigenous traditional medicines (e.g., Traditional Chinese Medicine, Ayurveda) while entirely failing to regulate the indigenous African Traditional Medicines on which most of its Black population depends.

Category D complementary medicines include discipline-specific medicines and health supplements. Discipline-specific medicines are those recognized and regulated by the SAHPRA in six main disciplines. The disciplines of Aromatherapy, Ayurveda, Homeopathy, Traditional Chinese Medicine, Unani Tibb, and Western Herbal Medicine are explicitly described by SAHPRA as traditional medicines that are not indigenous to South Africa (12). Yet an effective regulatory framework has been established. Conversely, ATM, indigenous to South Africa, lacks a framework, recognition, or registration procedure. Likewise, traditional medicines from other parts of Africa have no place in South Africa’s regulatory landscape.

This regulatory gap directly contradicts the Constitution’s Bill of Rights, which guarantees citizens the right to participate in their chosen cultural life and select their preferred healthcare (3). By regulating foreign traditional medicines while ignoring indigenous ones, the state implicitly privileges non-African medical systems over African ones. This is not merely an oversight but a structural injustice.

The registration requirements, ZA-CTD format, criteria for risk classification, and safety and efficacy documentation detailed in this study apply exclusively to Category D discipline-specific complementary medicines. Our findings reveal that, in the registration and sale of these non-indigenous medicines, quality, safety, and efficacy are critically considered, and their availability is aligned with their relative risk, as provided in Tables 1 and 2 (23, 29). However, no such considerations apply to ATM. We speculate that this could be due to the WHO’s hegemonic policy control over SAHPRA. As has been demonstrated in the literature, the WHO framework prioritizes specific types of data, e.g., clinical trials, genomic validation, etc. as the only legitimate basis for regulation (25). This could indirectly pressure national regulatory authorities like SAHPRA to emulate, thereby effectively dismissing indigenous medical systems that do not or cannot conform to these specific parameters.

### Identifying the regulatory gap in the current South African system

In summary, our study identified the regulatory deficit in the current South African system. The current regulatory gap is characterized by a “conspicuous absence” of a policy framework for indigenous medicine. While SAHPRA has explicitly expanded its mandate to regulate six disciplines of non-indigenous traditional medicines under “Category D,” it has failed to create any parallel sub-category for indigenous ATM (12). This constitutes an institutional irregularity. Furthermore, ATM has been excluded from the contemporary regulatory frameworks. The regulatory tools that ensure the safety of other products, such as mandatory quality controls, standardized labeling, and clinical or traditional evidence documentation, are systematically denied to the ATM sector (23, 29). Finally, the current requirement for the South African Common Technical Document (ZA-CTD) format is designed for pharmaceutical and modern complementary products. This system is incompatible with the existing standards of the ATM as it imposes a high requirement that fails to account for the unique nature of ATM production. Essentially, while SAHPRA maintains domestic sovereignty under the South African Constitution, the practical reality is a regulatory environment heavily influenced by WHO standards that prioritize conventional scientific validation (19, 25). This hegemonic influence makes it difficult to advance an indigenously developed regulatory pathway for ATMs without significant government investment in creating standardized evidence that meets global expectations (25), and this institutes constitutional and structural injustice.

### Proposed solution: a simplified and equitable regulatory pathway

To rectify this injustice, South Africa must adopt a practical and constitutionally sound approach. We are not arguing that ATMs should be subject to the exact same stringent requirements as conventional pharmaceuticals, which would be unrealistic and impracticable. Instead, we propose a simplified, layered, or risk-based model like those successfully implemented in other African nations (30).

Other African countries offer more equitable and practical models. A comparative study of locally manufactured herbal medicines in Ghana, Nigeria, Uganda, and Zimbabwe found that most offer simplified pathways before market authorisation. In Nigeria, locally manufactured herbal medicines are listed, whereas in Ghana and Uganda, they are notified. In Nigeria and Uganda, herbal products undergo two years of pharmacovigilance for post-marketing surveillance. Subsequently, in Uganda, they achieve full registration with the National Drug Authority (30). Lesotho has also introduced the Lesotho Medicines and Medical Devices Control Authority Act, 2023, to regulate traditional medicines and other medicines (31). The unique geographic, cultural, and socio-economic closeness between Lesotho and South Africa creates a strategic advantage for South Africa through SAPHRA to consider and learn from Lesotho’s Medicines and Medical Devices Control Authority Act, 2023. By learning from within the continent, South Africa could adopt a simplified, equitable registration pathway for locally manufactured herbal medicines, drawing on successful precedents from other African nations. These pathways are appropriate as they consider the uniqueness of ATMs:

• Simplified registration pathways could be implemented following the examples of Ghana, Nigeria, and Uganda. South Africa should introduce “notification” or “listing” systems that act as an entry-level regulatory framework, rather than applying the rigid, high-barrier requirements used for Category D medicines. These systems are practical as they work with the existing realities of the traditional medicine sector rather than imposing requirements that are impossible to meet.
• South Africa could implement a period of supervised post-marketing surveillance for locally manufactured herbal medicines similar to the models in Nigeria and Uganda. This allows for Pharmacovigilance to be conducted in phases whereby the collection of safety data will be conducted gradually while maintaining product availability, eventually leading to full registration.
• The regulatory framework should move away from a universal approach were same rules or solutions are used in every situation without considering factors to match unique circumstances (32). The evidence requirements must be balanced by legally recognizing traditional knowledge, history of long-term use, and ethnobotanical evidence as valid components of the safety and efficacy assessment for ATMs, consistent with their risk profile.
• SAHPRA must recognise the institution and expand its mandate to explicitly include a unit that is focused on indigenous ATM regulation. This approach ensures the involvement of traditional practitioners in the development of guidelines, thereby respecting cultural and religious beliefs, which are essential to the efficacy of the practice.

Implementing the above recommendations demonstrates a constitutional commitment of the state to protecting the rights of all its citizens (3). This sends a clear message that the health of the majority matters as much as the health of those who use non-indigenous systems. Previous studies have echoed the urgent need for an ATM regulatory framework (6, 18). The lack of such a framework has negative implications for public health, economic development, cultural rights and constitutional loyalty.

### Limitations

The primary limitation of our study is the lack of evidence for ATM regulation by the SAHPRA. Extensive resource materials and guidelines on complementary medicines are available on the SAHPRA website, which we have briefly summarised. However, the absence of evidence for ATM regulation is itself the central finding of this study.

## Conclusion

The status of the regulation and registration of African Traditional Medicines in South Africa is not merely unseemly; it is constitutionally unjustifiable. Although complementary medicines are traditional medicines, they are not ATMs. The complete absence of a regulatory framework for ATM, compared to the comprehensive framework for non-indigenous traditional medicines, constitutes a structural injustice that contradicts the Bill of Rights. This also poses public health risks to users. Effective integration of traditional medicine into South Africa’s health system can only be achieved when the regulation of traditional medicines is established and upheld in a manner that genuinely respects the cultural and religious beliefs of all South Africans, including, and especially, the majority who depend on African Traditional Medicines.
